# Effect of Vitamin D Supplementation on the Prognosis of Post-stroke Fatigue: A Retrospective Cohort Study

**DOI:** 10.3389/fneur.2021.690969

**Published:** 2021-11-05

**Authors:** Long Wang, Xue-min Zhao, Fu-yu Wang, Jun-Cang Wu, Yu Wang

**Affiliations:** ^1^Department of Neurology, The Second People's Hospital of Hefei, Hefei, China; ^2^Department of Neurology, General Hospital of Wan Bei Coal and Electrical Group, Suzhou, China; ^3^Department of Pharmacy, The Second People's Hospital of Hefei, Hefei, China; ^4^Department of Neurology, The First Affiliated Hospital of Anhui Medical University, Hefei, China

**Keywords:** post-stroke, fatigue, vitamin D, supplementation, outcome

## Abstract

**Objective:** We aimed to evaluate the effect of vitamin D supplementation in post-stroke fatigue (PSF) patients with vitamin D deficiency on fatigue symptoms and outcomes.

**Methods:** Patients with primary acute ischemic stroke (AIS) were recruited consecutively from July 2016 to June 2018. Post-stroke fatigue patients were screened out with the Fatigue Severity Scale (FSS) questionnaire, serum concentrations of 25-hydroxyvitamin D [25-(OH)-D] were assessed with enzyme-linked immunosorbent assay (ELISA), and neurological function was evaluated with FSS and modified Rankin Scale (mRS) scoring criteria. Post-stroke fatigue patients with vitamin D deficiency were divided into two groups: a study group in which patients received vitamin D supplementation (cholecalciferol, 600 IU/day) along with usual care, and a control group in which patients received usual care alone. At the end of 1 and 3 months after treatment, all PSE patients accepted re-measurement of serum vitamin D and re-evaluation of fatigue and neurological function.

**Results:** A total of 532 AIS patients were consecutively recruited to participate in this study. Patients without PSF, non-vitamin D deficiency, pre-stroke fatigue, or vitamin D supplementation were excluded from the study. In addition, patients who were lost to follow-up were also excluded. Finally, 139 out of 532 (26.1%) patients with PSF and vitamin D deficiency received vitamin D supplementation treatment. Fatigue Severity Scale score was significantly lower in the study group than in the control group at 1 month (*t* = −4.731, *p* < 0.01) and 3 months (*t* = −7.937, *p* < 0.01) after treatment. One month after treatment, mRS score in the study group was lower than that in the control group without statistical difference (*t* = −0.660, *p* > 0.05), whereas mRS was significantly higher in the study group than in the control group at 3 months after treatment (*t* = −4.715, *p* < 0.01).

**Conclusions:** Our results indicated that vitamin D supplementation could improve fatigue symptoms and neurological outcomes in PSF patients with vitamin D deficiency. Subject to replication in other settings, a randomized controlled trial (RCT) might be undertaken to validate the potential beneficial impact of vitamin D supplementation in post-stroke patients found to be vitamin D deficient.

## Introduction

Post-stroke fatigue (PSF) is often overlooked though it is common in patients after stroke. The estimated prevalence of PSF ranges from 23 to 75%, and its symptoms may continue up to 1 year or more (1–3). Post-stroke fatigue has been defined as pathological tiredness unrelated to intensity of exercise in the post-stroke period. Apart from distress reported by PSD patients, PSF has been found to be related to the increase of mortality (4). Although the etiology of PSF remains unclear, it is reported that the occurrence of PSF is associated with several predisposing factors such as vitamin D deficiency, depression, and premorbid fatigue (5–7).

In humans, vitamin D, a fat-soluble hormone, is supplied from different sources such as skin exposure to light (8), food, or by supplementation. Vitamin D is converted to 25-hydroxyvitamin D [25-(OH)-D] in the liver by different 25-hydroxylase enzymes. Measurement of serum level of 25-(OH)-D is used to reflect the level of vitamin D as it best reflects vitamin D supply from all different sources (9). In recent years, studies have found that vitamin D level not only plays a role in regulating calcium and phosphorus metabolism but also has association with the severity and prognosis of stroke (10–12). Vitamin D has a protective effect on endothelial function, vascular remodeling, and neuromuscular and immunological functions in experimental models (13, 14). Additionally, recent lines of evidence have shown a close link between vitamin D deficiency and fatigue occurrence in both healthy and clinical conditions (15–17).

One meta-analysis had shown that vitamin D deficiency was related with 2.5-fold increase of the risk of acute ischemic stroke (AIS) (18). Another meta-analysis involving 28,072 patients suggested that calcium supplementation, with or without vitamin D deficiency, could modestly increase the risk of stroke (19). It is noteworthy that these studies were aimed at primary prevention of stroke but not at post-stroke conditions. The benefit of vitamin D supplementation in improving outcomes in AIS patients remains controversial (20, 21).

Considering the involvement of vitamin D in fatigue and outcomes in AIS, we retrospectively analyzed the efficacy of vitamin D supplementation in fatigue and neurological outcome improvement in PSF patients with vitamin D deficiency through 3 months' follow-up.

## Methods

### Study Design

We conducted a retrospective study to assess the effectiveness of vitamin D supplementation on the prognosis of PSF in stroke patients admitted to the Department of Neurology, General Hospital of Wan Bei Coal and Electrical Group from July 2016 to June 2018.

This study was approved by the General Hospital of Wan Bei Coal and Electrical Group Research Ethics Board and without the need for informed consent by using the medical data of the participants.

### Inclusion and Exclusion Criteria

Fatigue was examined using the Fatigue Severity Scale (FSS), which was proved to be valid and reliable in various clinical groups (22, 23). The FSS contains seven items where higher mean scores reflect higher degrees of fatigue (24). Fatigue was defined as mean FSS score ≥4 (23).

All enrolled patients with AIS were confirmed by 3.0 T cranial magnetic resonance imaging (MRI) with the mean FSS scored above four points. Inclusion criteria were as follows: (1) aged between 40 and 75 years old; (2) first-ever stroke with MRI scan upon admission and confirmed AIS; and (3) FSS score was evaluated at 1 week after onset.

The exclusion criteria were as follows: (1) diagnosed with transient ischemic attack; (2) previous stroke or any other central nervous system disease, such as brain trauma, tumor, and cerebritis; (3) fatigue within 1 month before admission; (4) severe liver and kidney dysfunction; (5) taking vitamin D or other drugs affecting bone metabolism within 3 months; and (6) cognitive or speech disorders that may affect evaluation.

### Data Collection

The patients' demographic characteristics including gender and age were obtained from their medical records. Baseline clinical parameters were collected, including stroke subtype (based on the TOAST criteria), current smoking, and arterial blood pressure. Pre-existing comorbidities included hypertension, diabetes, and coronary heart disease (CHD). The baseline laboratory tests, including 25-(OH)-D, fasting blood glucose (FBG), low-density lipoprotein cholesterol (LDL-C), total cholesterol (TC), and uric acid (UA), were obtained within 24 h of admission. Additionally, the stroke severity was assessed by experienced neurologists according to the NIHSS within 24 h of admission.

### Serum 25-(OH)-D Assessment and Treatment

Venous blood samples were collected in plain biochemical tube within 24 h of admission. Serum 25-(OH)-D concentration was measured by chemiluminescent immunoassay (Cobase 6000, Roche Diagnostics, Swiss). Similar to previous studies, a normal serum level of vitamin D was defined as 25-(OH)-D concentration >30 ng/ml (25). Vitamin D deficiency was defined as 25-(OH)-D concentration <20 ng/ml (25, 26).

Study group: patients with vitamin D deficiency in the study were treated with combination therapy of vitamin D_3_ ® (cholecalciferol, 600 IU/day) for 3 months.

Control group: patients with vitamin D deficiency were not treated with combined vitamin D due to out-of-pocket payment or lack of cooperation.

In addition, all enrolled patients were treated in accordance with the American Heart/Stroke Association Guidelines for the Management of Acute Ischemic Stroke (2018) (27). Individual treatment was planned based on the condition of each patient.

### Follow-Up Records

The mRS is a graded interval scale [range, 0 (no symptoms) to 6 (death)] for the assessment of neurological function (28).

Serum 25-(OH)-D levels, FSS, and mRS scores were obtained from their follow-up records with re-measurement and re-evaluation at 1 and 3 months after treatment.

### Statistical Analysis

Continuous variables were described as mean (± standard deviation) for normally distributed data and median (four percentile) (25th and 75th interquartile range) for non-normally distributed data. Categorical variables were presented as numbers (percentages). The paired *t*-test for two means (paired observations) were applied for analyzing within-group changes from baseline in quantitative parameters. The repeated measure test was applied for analyzing the differences in categorical parameters between the study groups. Kruskal–Wallis test was used for statistical analysis of non-normal distribution data among groups. Pearson's correlations (*r*) were conducted to analyze the relationship between FSS scores and 25-(OH)-D levels. All analyses were carried out using SPSS (version 17.0). A *p*-value < 0.05 was considered to be statistically significant.

## Results

### Study Process

**Figure 1:**
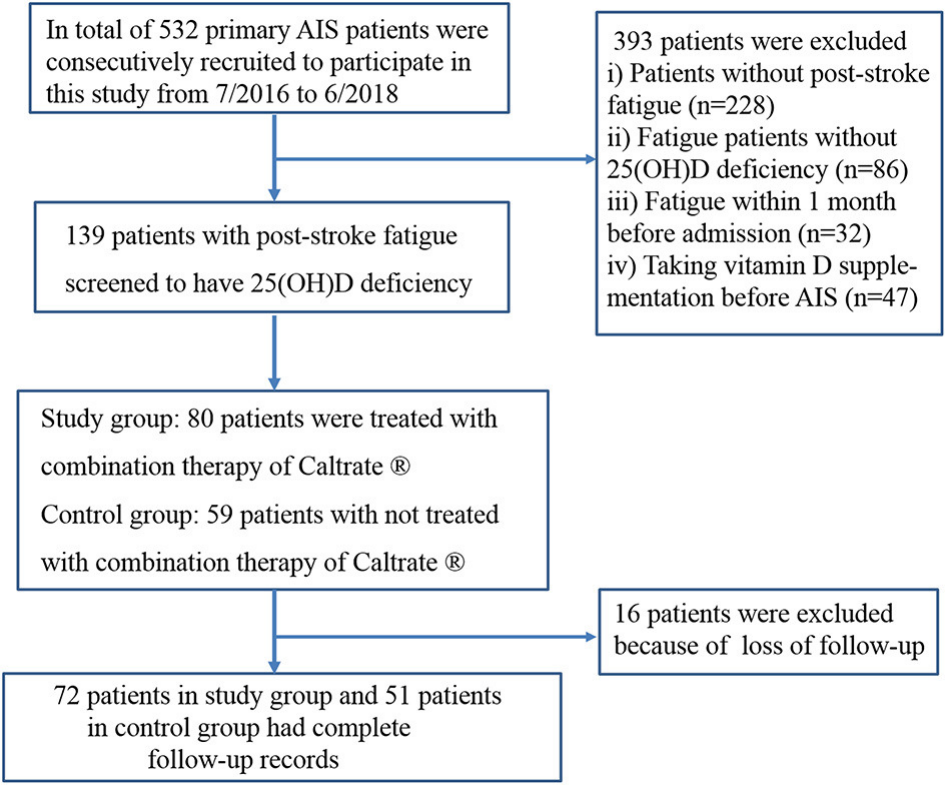
The study flow chart.

As illustrated in the study flow in Figure 1, a total of 139 patients met all inclusion criteria and screened to have vitamin D deficiency. Seventy-two patients in the study group and 51 patients in the control group had complete follow-up records (Figure 1).

### Baseline Characteristics of Patients

Two groups did not differ in respect to baseline characteristics, including age, gender, clinical parameters, pre-existing comorbidities, and biochemical variables. The demographics and characteristics of the patients are displayed in Table 1.

**Table 1:** The demographic and clinical aspects of the study population.

	**Study group**	**Control group**	***t*** **/χ^2^**	***P***
	**(***n*** = 72)**	**(***n*** = 51)**		
Age (±SD), years	62.0 ± 8.2	64.4 ± 8.6	−1.418	0.159
Male, *n* %	30 (41.7)	29 (56.9)	2.762	0.097
Hypertension, *n* (%)	40 (55.6)	32 (62.7)	0.636	0.425
Diabetes, *n* (%)	25 (34.7)	14 (27.5)	0.729	0.393
CHD, *n* (%)	2 (2.8)	3 (5.9)	0.738	0.390
Hyperlipidemia, *n* (%)	43 (59.7)	22 (45.1)	3.295	0.069
Current smoking, *n* (%)	33 (45.8)	17 (33.3)	1.933	0.164
Stroke subtype, *n* (%)			1.456	0.692
LAA	41 (56.9)	26 (51.0)		
SVO	28 (38.9)	24 (47.0)		
CE	2 (2.8)	1 (2.0)		
SOE and SUE	1 (1.4)	0		
IV thrombolysis	4 (5.6)	1 (2.0)	0.989	0.320
NIHSS score	5.8 ± 2.5	5.1 ± 2.2	1.479	0.142
FSS score	5.4 ± 1.4	5.4 ± 1.2	0.161	0.872
FBG, mmol/L	6.8 ± 1.9	6.5 ± 2.0	0.880	0.380
TC, mmol/L	4.7 ± 1.0	4.4 ± 0.9	1.827	0.070
LDL, mmol/L	2.8 ± 0.9	2.6 ± 0.6	1.278	0.204
UA, mmol/L	317.9 ± 89.2	297.0 ± 82.1	1.318	0.190
25-(OH)-D, ng/ml	13.1 ± 3.6	13.9 ± 4.3	−1.323	0.188

*CHD, coronary heart disease; LAA, large artery atherosclerosis; SVO, small vessel occlusion; CE, cardioembolism; SOE, stroke of other determined etiology; SUE, stroke of undetermined etiology; NIHSS, National Institute of Health Stroke Scale; IV, intravenous; TC, total cholesterol; HDL, high-density lipoprotein; LDL, low-density lipoprotein; FBG, fasting blood glucose; UA, uric acid*.

### Outcomes

**Figure 2:**
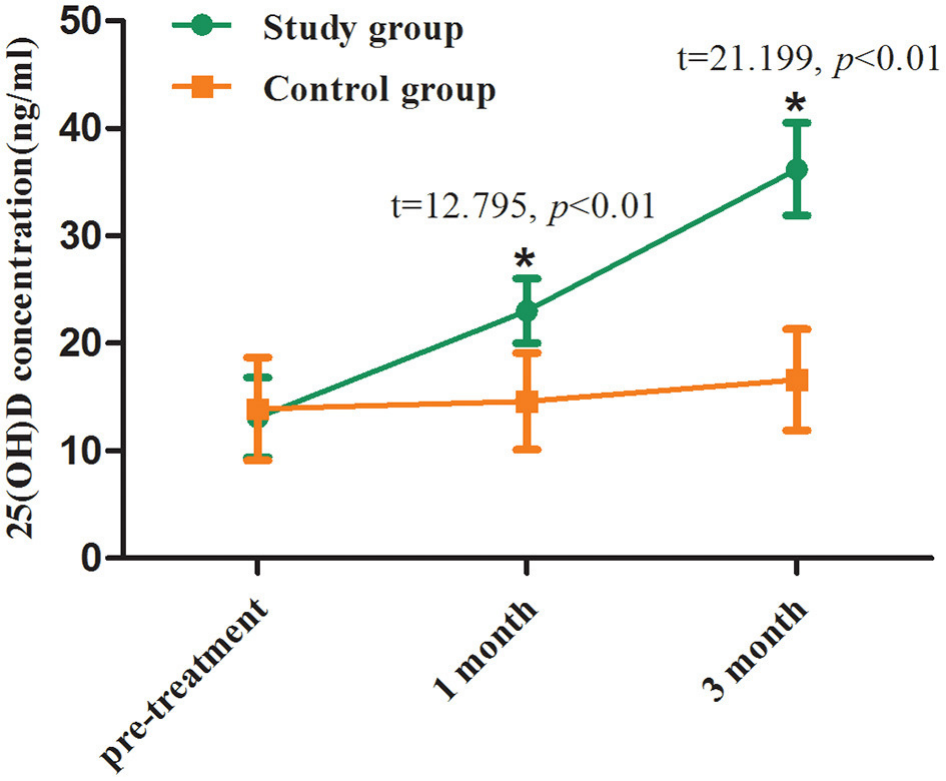
Serum 25-(OH)-D levels before and after treatment between two groups. Comparing to the control group, ^*^p < 0.01.

The 25-(OH)-D levels before supplement in the two groups were 13.1 ± 3.6 and 13.9 ± 4.3 ng/ml, respectively, with no significant difference (*p* > 0.05). At the first and third month of follow-up, the mean 25-(OH)-D levels in the study group were 22.8 and 35.7 ng/ml, respectively, which were significantly higher than those at the corresponding time points in the control group (*p* < 0.01, Table 2; Figure 2).

**Table 2:** Serum 25-(OH)-D levels before and after treatment between two groups.

**Time**	**Study group**	**Control group**	***t***	***P***
Pre-treatment	13.1 ± 3.6	13.9 ± 4.3	−1.323	0.188
Follow-up (1 month)	22.8 ± 3.2	14.2 ± 4.0	12.795	<0.001
Follow-up (3 month)	35.7 ± 5.2	16.5 ± 4.5	21.199	<0.001

**Figure 3:**
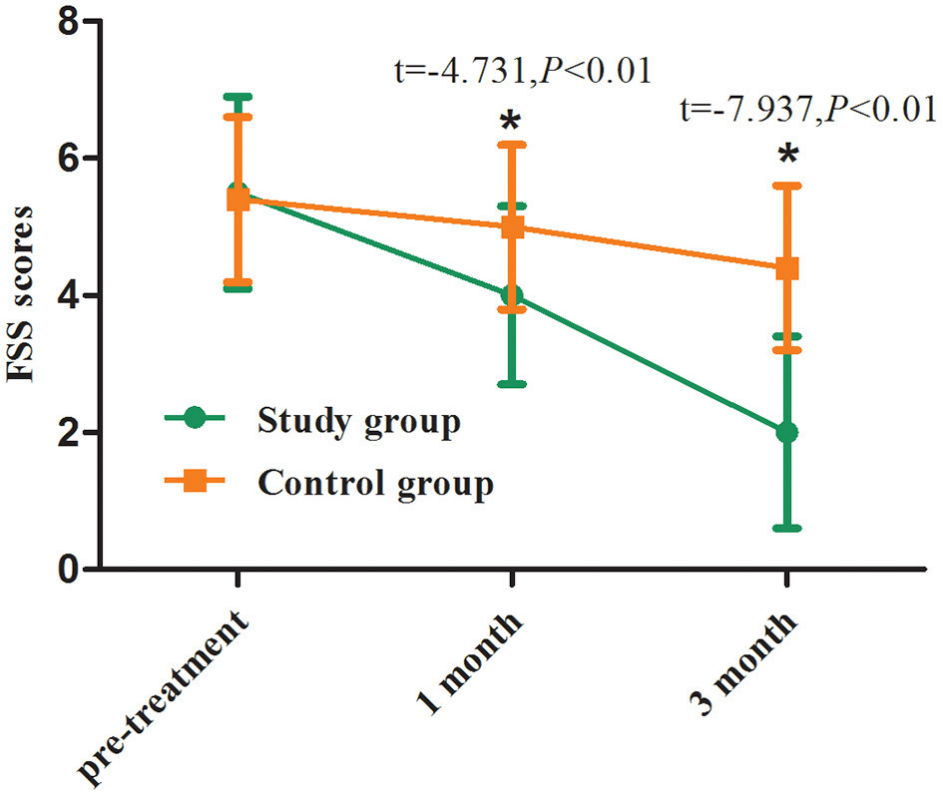
FSS scores before and after treatment between two groups. Comparing to the control group, ^*^p < 0.01.

The FSS scores before vitamin D_3_ supplementation in the two groups were 5 [4, 7] and 6 [4, 6] ng/ml, respectively, with no significant difference (*p* > 0.05). At the first and third month of follow-up, the mean FSS scores in the study group were 4.0 and 2.0 ng/ml, respectively, which was significantly lower than those at the corresponding time points in the control group (*p* < 0.01, Table 3; Figure 3).

**Table 3:** Neurological symptom scoring before and after treatment between two groups.

**Variable**	**Study group**			**Control group**		
	**Pre-treatment**	**Follow-up (1month)**	**Follow-up (3month)**	**Pre-treatment**	**Follow-up (1month)**	**Follow-up (3month)**
FSS, scores	5 [4, 7]	4 [3, 5]	2 [1, 3]	6 [4, 6]	5 [4, 6]	4 [3, 5]
mRS, scores	3 [2, 3]	2 [1, 3]	1 [1, 2]	3 [3, 3]	2 [2, 3]	2 [1, 2]

**Figure 4:**
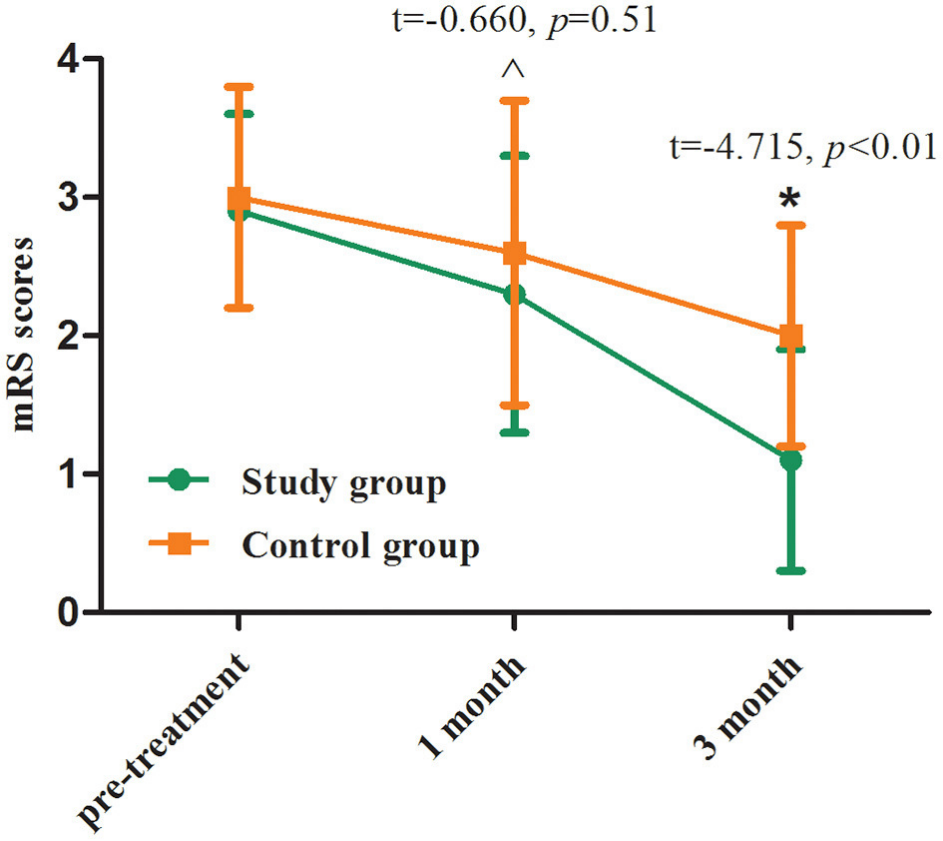
mRS scores before and after treatment between two groups. Comparing to the control group, ^∧^*p* > 0.05, ^*^p < 0.01.

One month after treatment, mRS score in the study group was lower than that in the control group without statistical difference (*p* > 0.05), whereas mRS was significantly higher in the study group than in the control group at 3 months after treatment (*p* < 0.01, Table 3; Figure 4).

Correlation analysis showed that FSS scores were negatively correlated with serum 25-(OH)-D levels (*r* = −0.65, *p* < 0.001).

## Discussion

Previous studies have demonstrated that a substantial proportion of stroke patients are afflicted with vitamin D deficiency associated with AIS severity, PSF, and worse functional outcomes (29–32). However, there are few trial studies evidencing whether vitamin D supplementation has a positive effect on the stroke outcome (11, 33).

To the best of our knowledge, the current study reports, for the first time, a dynamic evaluation of the effect of vitamin D supplementation on PSF and outcomes. After 1 month of vitamin D supplementation, the fatigue scores in the study group were significantly lower than those of the control group. Nevertheless, we did not find a difference in mRS scores between the two groups, but observed a trend toward decrease in mRS. With 25-(OH)-D level increased, a significant difference in mRS scores was observed between two groups with better neurological outcome achieved in the study group at 3 months after supplementation. Our finding may provide a potential proposal for the treatment of fatigue and functional deficits in AIS.

The prospective non-randomized studies have shown that normalization of vitamin D concentration could improve fatigue in patients with various medical conditions (34–36). Our findings are in keeping with a recent randomized controlled trial (RCT) showing that a single intramuscular injection of vitamin D_3_ (600,000 IU) improved Scandinavian Stroke Scale (SSS) score in vitamin D-deficient patients at 12 weeks following AIS (21). However, another recent RCT suggested that oral vitamin D supplementation (2,000 IU/day) does not improve rehabilitation outcomes after AIS (37). These research discrepancies may be caused by a variety of mixed factors, such as supplemental dosage and mode of vitamin D, environmental and social factors for the study subjects, study design, and outcome measures in each trials.

The mechanisms of vitamin D against PSF remain unclear to date, but several neuroprotective mechanisms have been proposed. Vitamin D deficiency induces muscle weakness and myalgia especially in stroke patients with neurological impairment (38, 39). Adequate vitamin D level promotes the expression of insulin-like growth factor 1, which has neuroprotective ability against axonal and dendrite degeneration (39, 40), as well as antithrombotic capabilities by activating plasminogen (41). Another possible explanation is that vitamin D is involved in inhibiting inflammation and oxidative stress, increasing post-stroke blood flow to central nervous system through enhancing the activity of nitric oxide synthase (42, 43). Furthermore, vitamin D therapy improves muscle mitochondrial oxidative phosphorylation that potentially causes a modulation of fatigue (44).

Previously tried drug interventions including the use of Fluoxetine and Modafinil have been shown largely ineffective for PSF (45, 46). Future longitudinal RCT with a larger sample size confirming our findings may provide a novel therapeutic option to improve the prognosis of PSF.

Several limitations of this study should be considered. Firstly, the limited number of subjects in our study reduced the statistical power. Secondly, other confounders such as nutritional habits and lifestyle that may affect serum level of 25-(OH)-D (47) were not assessed in this study. Thirdly, the main shortcoming of this intervention study was the lack of a placebo-control group as it was a retrospective study. Thus, a minimum replication study or, more ideally, a prospective cohort study is needed before any RCT is considered. Despite these limitations, our findings suggested an association between vitamin D level and outcomes in patients with PSF and a therapeutic option may be recommended for PSF patients.

## Conclusion

Taken together, our results indicated that vitamin D supplementation could improve fatigue symptoms and outcomes in PSF patients with vitamin D deficiency. Subject to replication in other settings, RCT might be undertaken to validate the potential beneficial impact of vitamin D supplementation in post-stroke patients found to be vitamin D deficient.

## Data Availability Statement

The original contributions presented in the study are included in the article/supplementary material, further inquiries can be directed to the corresponding author/s.

## Ethics Statement

The studies involving human participants were reviewed and approved by General Hospital of Wanbei Coal and Electrical Group Research Ethics Board. Written informed consent for participation was not required for this study in accordance with the national legislation and the institutional requirements.

## Author Contributions

The study was conceived by LW, J-CW, and YW. Case collection and data compilation were conducted by LW and X-mZ. Follow-up was performed by F-yW, LW, and X-mZ. The rough manuscript was drafted by LW and YW. All authors reviewed and approved the final version of the manuscript.

## Funding

This study was supported by the Beng Bu Medical College Project (2017BYKY17191) and the National Natural Science Foundation of China (81671290).

## Conflict of Interest

The authors declare that the research was conducted in the absence of any commercial or financial relationships that could be construed as a potential conflict of interest.

## Publisher's Note

All claims expressed in this article are solely those of the authors and do not necessarily represent those of their affiliated organizations, or those of the publisher, the editors and the reviewers. Any product that may be evaluated in this article, or claim that may be made by its manufacturer, is not guaranteed or endorsed by the publisher.
